# Effectiveness of Music Therapy for Delirium in Acute Hospital Settings: A Scoping Review

**DOI:** 10.3390/nursrep16010023

**Published:** 2026-01-12

**Authors:** Stacey Leonard, Elizabeth Henderson, Gary Mitchell

**Affiliations:** School of Nursing and Midwifery, Queen’s University Belfast, Belfast BT9 7BL, UK; sleonard04@qub.ac.uk (S.L.); gary.mitchell@qub.ac.uk (G.M.)

**Keywords:** music therapy, delirium, acute hospital care, non-pharmacological interventions, older adults, behavioural symptoms, postoperative delirium, scoping review

## Abstract

**Background**: Music therapy is a non-pharmacological psychosocial intervention that is increasingly recognised for its role in supporting older adults in acute hospital settings. Engagement with music, whether through passive listening, preferred recorded music, live music, or creative music therapy, has been linked to improvements in behavioural, cognitive, and emotional outcomes during episodes of delirium. Although there are reviews on non-pharmacological approaches to delirium, few have focused specifically on music therapy within acute hospital environments. **Methods**: This scoping review examined the evidence relating to music-based interventions for older adults who are experiencing delirium or who are at risk of delirium in acute care settings. The review followed the Preferred Reporting Items for Systematic Reviews and Meta-Analyses extension for Scoping Reviews (PRISMA ScR). Four electronic databases were searched systematically, namely, CINAHL, Medline, PsycINFO and Embase. **Results**: Seven primary research studies published between 2004 and 2024 met the inclusion criteria. A narrative synthesis approach was used to summarise the data. Three themes were identified. The first relates to the extent to which music therapy may reduce the incidence or severity of delirium or other related behaviours in acute hospital settings. The second relates to the potential for music-based interventions to support clinical practice by improving interaction between patients and staff and reducing distress during recovery and enhancing physical recovery. The third relates to the impact of music therapy on emotional regulation, engagement, cooperation with care, and overall patient experience. **Conclusion**: Music therapy shows promise as a person-centred, safe, and low-cost intervention that may enhance wellbeing and support delirium care for older adults in acute hospital settings. Further high-quality studies are needed to strengthen the evidence base and guide practice.

## 1. Introduction

Delirium is an acute neuropsychiatric syndrome characterised by a rapid onset, fluctuating trajectory, and disturbances in cognition, perception, behaviour, and consciousness. It presents across hyperactive, hypoactive, and mixed motoric subtypes, each associated with different clinical manifestations and care implications. Contemporary guidelines, including those from NICE [1], emphasise that delirium is not a discrete disease but a multifactorial syndrome resulting from complex interactions between vulnerable patient characteristics and acute physiological stressors. Underlying mechanisms remain incompletely understood, although dysregulated neurotransmission, neuroinflammation, metabolic disturbance, and disruption of neural connectivity are all thought to contribute to its pathophysiology [2]. This complexity underscores the necessity for interdisciplinary, person-centred clinical approaches.

Recent literature reflects a growing recognition of delirium as a global health priority due to its prevalence, clinical burden, and preventable nature. A worldwide study identified a delirium prevalence of 18% among general hospital inpatients, with markedly higher rates of 31–54% in intensive care settings [3,4]. Its presence is consistently associated with increased mortality, prolonged hospitalisation, institutionalisation, and long-term functional and cognitive decline [5]. Moreover, in-hospital delirium generates substantial economic consequences, with estimated additional costs ranging between USD 806 and USD 24,509 per admission. Despite this well-documented burden, delirium remains systematically under-recognised and under-reported in clinical practice [6]. Concerningly, even with increasing research on delirium screening, structured guidelines, and validated tools, clinical staff frequently report knowledge deficits and limited training in delirium identification and management. Grover et al. [7] found that 92.8% of nurses surveyed had never received education specific to delirium care, highlighting a persistent implementation gap.

Delirium also exerts profound psychological effects on patients and their families. Caregivers often experience emotional strain, fear, and distress when witnessing sudden cognitive and behavioural changes [6]. People living with dementia are at particular risk, with more than half developing delirium during hospitalisation [8]. Delirium superimposed on dementia (DSD) exacerbates existing cognitive impairment, further impairs function, and frequently goes unrecognised due to overlap with baseline cognitive deficits [9]. DSD is associated with longer hospital stays, greater dependency in activities of daily living, and increased mortality [10]. These intersections between delirium, dementia, and frailty highlight the critical need for early detection, personalised interventions, and integrated multidisciplinary care.

Clinically, delirium has been described for centuries, with roots tracing back to Hippocrates [11]. Modern conceptualisation distinguishes hyperactive, hypoactive, and mixed presentations [12]. Hyperactive delirium, characterised by agitation, hallucinations, restlessness, and aggression, often draws rapid clinical attention because of its disruptive and unsafe behaviours [13]. Hypoactive delirium, however, is more prevalent yet frequently overlooked due to its subtle features such as lethargy, reduced motor activity, and withdrawn behaviour [14]. Mixed delirium involves fluctuating transitions between hyperactivity and hypoactivity, creating additional diagnostic challenges and often indicating multiple precipitating factors [15]. Given this heterogeneity, person-centred assessment and management approaches are essential and align with frameworks such as Kitwood’s person-centred care model [16] and McCance and McCormack’s Person-Centred Nursing Framework [17].

Risk factors for delirium are generally categorised into predisposing vulnerabilities and precipitating insults [18]. Approximately 30–40% of inpatient delirium cases are preventable [19], positioning delirium prevention as a core patient-safety priority. Common predisposing factors include advanced age, dementia, sensory impairment, functional decline, frailty, and chronic illness. Acute precipitants include infection, dehydration, hypoxia, surgery, pain, sleep disturbance, and polypharmacy. Failure to identify and address modifiable risk factors increases vulnerability to complications such as aspiration pneumonia, pressure ulcers, falls, malnutrition, prolonged dependency, and long-term cognitive impairment [20].

Timely diagnosis requires a comprehensive history, clinical examination, and structured assessment using validated tools such as the Confusion Assessment Method (CAM), the CAM-ICU, the 4AT, or the Nursing Delirium Screening Scale (Nu-DESC). Collateral history remains indispensable, particularly when patients are unable to reliably report their symptoms or baseline cognitive function [21]. Despite the availability of validated instruments, barriers such as communication impairments, fluctuating arousal, and pre-existing cognitive deficits continue to hinder accurate assessment [22].

Management of delirium requires a multifaceted, person-centred strategy addressing underlying causes including infection, dehydration, constipation, pain, urinary retention, and medication-related contributors. While antipsychotic medication may be considered for severe agitation posing risk to patient or staff safety, evidence does not support their routine use, and treatment should always be time-limited and carefully monitored [1,23]. Sleep promotion, optimisation of sensory input, mobility, and environmental stabilisation are universally recommended due to their strong evidence base and low risk.

Growing attention has been directed toward non-pharmacological interventions, which are considered first-line treatments due to their safety and alignment with the principles of regional Mental Capacity Acts. Multi-component interventions including reorientation, sensory optimisation, sleep hygiene, mobilisation, and hydration can reduce the incidence of delirium by up to 43% [24]. Family engagement is increasingly recognised as a powerful asset in delirium detection and management, with relatives often noticing subtle cognitive or behavioural changes earlier than clinical staff [25]. Their involvement can reduce the delirium duration and support patient reassurance and emotional stability [26].

Music therapy or structured music listening intervention represents a particularly promising non-pharmacological strategy. Emerging evidence suggests that music, whether through passive listening or structured music therapy sessions, can reduce delirium incidence, alleviate anxiety, and improve emotional regulation [27,28]. Its non-invasive, low-cost profile makes it well suited to older and cognitively impaired populations. However, inappropriate or overstimulating music may trigger distress in some individuals, emphasising the importance of personalised, culturally sensitive selection guided by patient preference and family insight [29,30]. This further highlights the importance of involving a trained music therapist, who is equipped to assess individual sensory thresholds and clinical context and is therefore less likely to select inappropriate or overstimulating music for patients experiencing delirium.

In response to the multifactorial nature of delirium, many healthcare systems have implemented delirium care bundles derived from NICE [1] guidance. Although no universal NHS-wide standardised bundle exists, most incorporate components addressing hydration, nutrition, mobility, pain, infection, and sleep. Their implementation aims to reduce incidence, mitigate severity, and improve overall patient outcomes [31].

Despite significant progress in understanding delirium, substantial gaps remain in practice, particularly in consistent implementation of evidence-based non-pharmacological approaches and in tailored strategies for specific populations, such as those with dementia or those in acute surgical or intensive care settings. These gaps highlight the need for further research, particularly on interventions such as music therapy, which show promise but remain under-evaluated across diverse clinical contexts. The growing interest in personalised, sensory-focused, and patient-centred strategies offers important opportunities to address unmet needs and enhance the quality of delirium care. Unlike existing reviews that predominantly focus on intensive care unit or mixed care settings, this scoping review specifically examines music therapy and music-based interventions for delirium within acute medical and surgical hospital wards, where evidence remains comparatively limited.

Given the ageing population, the high prevalence of delirium in acute hospital settings, and limited pharmacological options, there is a clear need to synthesise evidence on music therapy for delirium care. Understanding how music interventions have been implemented, which populations have been studied, and how outcomes have been measured is essential to inform practice, education, and future research. Unlike existing reviews that focus predominantly on intensive care or mixed settings, this scoping review specifically maps the use of music therapy and music-based interventions within non-ICU acute medical and surgical wards, clarifying the scope, characteristics and reported outcomes of the current evidence base rather than evaluating intervention efficacy.

A scoping review methodology was employed to map heterogeneous study designs, interventions, and outcomes, clarify key concepts, and identify gaps in the literature [32]. This review examines evidence on the prevention and management of delirium among adults in non-ICU acute hospital settings, including postoperative populations, with a focus on music therapy and structured music-based interventions as the primary exposure. It aims to describe study characteristics, explore delirium-related and patient-centred outcomes, and highlight implications for acute care practice and future research.

## 2. Materials and Methods

### 2.1. Study Design

This scoping review followed a structured and transparent methodological approach. The review procedures were guided by the Joanna Briggs Institute (JBI) framework for evidence synthesis [33], the foundational methodological guidance proposed by Arksey and O’Malley [32], and subsequent enhancements to scoping review methods by Levac and colleagues [34]. In addition, the reporting structure aligns with the Preferred Reporting Items for Systematic Reviews and Meta-Analyses extension for Scoping Reviews (PRISMA-ScR) [35].

### 2.2. Search Method

Four electronic databases were systematically searched in October 2024: CINAHL, MEDLINE (via Ovid), PsycINFO, and Embase. These databases were selected because they offer comprehensive coverage of nursing, medical, psychological and allied health research relevant to delirium, acute hospital care and music-based interventions. Together, they provided a robust platform for identifying interdisciplinary evidence applicable to adults experiencing delirium within acute hospital settings.

The search strategy was developed following an initial exploratory scan of the literature and refined to align with the primary objective of this scoping review. Search terminology was informed by previous reviews examining non-pharmacological interventions for delirium and music therapy in clinical settings, as well as the keywords used in delirium epidemiology and intervention studies. Broader music-related terms were intentionally included in the search strategy to ensure comprehensive capture of studies relevant to music therapy practice, recognising that music therapy is frequently mislabelled or inconsistently described in the literature. The search strategy was reviewed collaboratively by a subject librarian and two researchers experienced in scoping review methodology to ensure completeness and precision.

Search terms included MeSH terms and free-text keywords, structured around two core concepts: delirium and music therapy. Boolean operators (AND, OR) were used to combine and expand terms. The search included variations in terminology commonly used in acute hospital delirium research. The final search string included combinations of the following: Delirium OR Acute Confusion OR Confusion OR Mental Confusion OR Altered Mental Status AND Music Therapy OR Musical Intervention OR Music Based Therapy OR Music Treatment OR Music Assisted Therapy OR Sound Therapy.

Filters were not applied for year limits in order to capture the full scope of the literature, although all included studies were screened manually for relevance to adult acute care settings. Reference list searching of included studies was also undertaken to identify additional eligible papers not captured in the initial database search. All search results were exported into EndNote™ (Thomson Reuters™, Toronto, ON, Canada), where duplicates were automatically identified and removed. The remaining records were then transferred to a screening spreadsheet to facilitate independent and transparent title, abstract, and full-text screening. Searches were conducted in October 2024, using both controlled vocabulary and free-text terms adapted for each database, with title and abstract fields searched. Broader delirium-related terms were included to maximise sensitivity, and music-related terms were mapped to relevant subject headings where available. Full database-specific search strategies are provided in Supplementary File S1.

### 2.3. Inclusion and Exclusion Criteria

The Population–Exposure (PE) framework was used to develop inclusion criteria, in line with scoping review guidance [33,34]. This approach supported comprehensive mapping of studies examining the therapeutic use of music in relation to delirium across acute hospital settings, recognising the heterogeneity of study designs, interventions and outcome measures. Consistent with scoping review methodology, the focus was on describing concepts, interventions and contexts rather than evaluating effectiveness. All empirical study designs and evidence reviews were eligible, including quantitative, qualitative and mixed-methods research, with the PE framework enabling broad mapping of how outcomes and effectiveness have been examined across the literature. The review was limited to studies published within the last ten years to ensure relevance to contemporary delirium care practices and acute hospital contexts, and ICU-based studies were excluded because delirium management and care environments in intensive care units differ substantially from those in acute medical and surgical wards, which were the focus of this review. In keeping with scoping review methodology, the term “effectiveness” is used throughout to reflect how included studies reported outcomes related to music-based interventions, rather than to imply formal evaluation or comparison of intervention efficacy.

No geographical limits were applied. Studies were eligible if they focused on adults (≥18 years) in acute hospital settings where music therapy or structured music interventions were used in relation to delirium or acute confusional states. Studies were excluded if they were conducted in an ICU or non-acute settings (e.g., long-term care, community, rehabilitation), if delirium could not be distinguished from pre-existing dementia, or if music was part of a multicomponent intervention where its specific effects could not be isolated.

### 2.4. Data Extraction

First-level screening involved review of titles and abstracts by two reviewers (EH and SL). Preliminary exclusions removed studies that did not relate to music therapy or structured music interventions, those not addressing delirium or acute confusional states, studies involving paediatric populations, and non-English publications. Full-text papers were then reviewed to determine eligibility. Exclusion criteria at this stage included: studies conducted outside acute hospital settings, papers where delirium could not be distinguished from baseline dementia, multicomponent interventions where the specific contribution of music could not be isolated, non-empirical papers, dissertations, pre-2014 publications, and non–peer-reviewed reports.

After full-text screening, seven studies met the criteria for inclusion. A final screening stage involved hand-searching reference lists of all included articles and cross-checking citations in Google Scholar. No additional studies met the inclusion criteria. All Stage 1 and Stage 2 screenings were completed independently by two reviewers (EH and SL). A third reviewer (GM) was consulted when uncertainties regarding inclusion or exclusion arose.

### 2.5. Narrative Synthesis

Data were analysed using a narrative synthesis approach, which supports the examination of relationships within and across studies by organising findings into meaningful categories to identify recurring patterns [36,37,38,39,40,41,42]. Narrative synthesis is particularly suited to scoping reviews where included studies vary in design, methodology and outcomes, as is the case with research on music therapy for delirium in acute hospital settings. This approach allows the integration of quantitative, qualitative and mixed-methods evidence to build a coherent account of the available literature. To enhance transparency and rigour, SL maintained a reflective journal documenting analytical decisions, emerging interpretations and considerations of potential bias. This process supported ongoing reflexivity and strengthened the credibility of the synthesis.

The synthesis proceeded in three phases. Phase 1 involved structured data extraction using a standardised template to ensure consistent capture of study characteristics, intervention details and delirium-related outcomes. Phase 2 consisted of inductive thematic analysis, where recurrent concepts were coded and grouped across the included studies. Phase 3 involved refining these categories into overarching descriptive themes, generating an integrated narrative of how music therapy has been implemented and evaluated in the management of delirium within acute hospital settings [36,37,38].

## 3. Results

The search yielded 145 records after removal of duplicates. Following title and abstract screening, 49 full-text papers were assessed for eligibility. After full-text review, seven studies met the inclusion criteria and were included in this scoping review [36,37,38,39,40,41,42].

**Figure 1:**
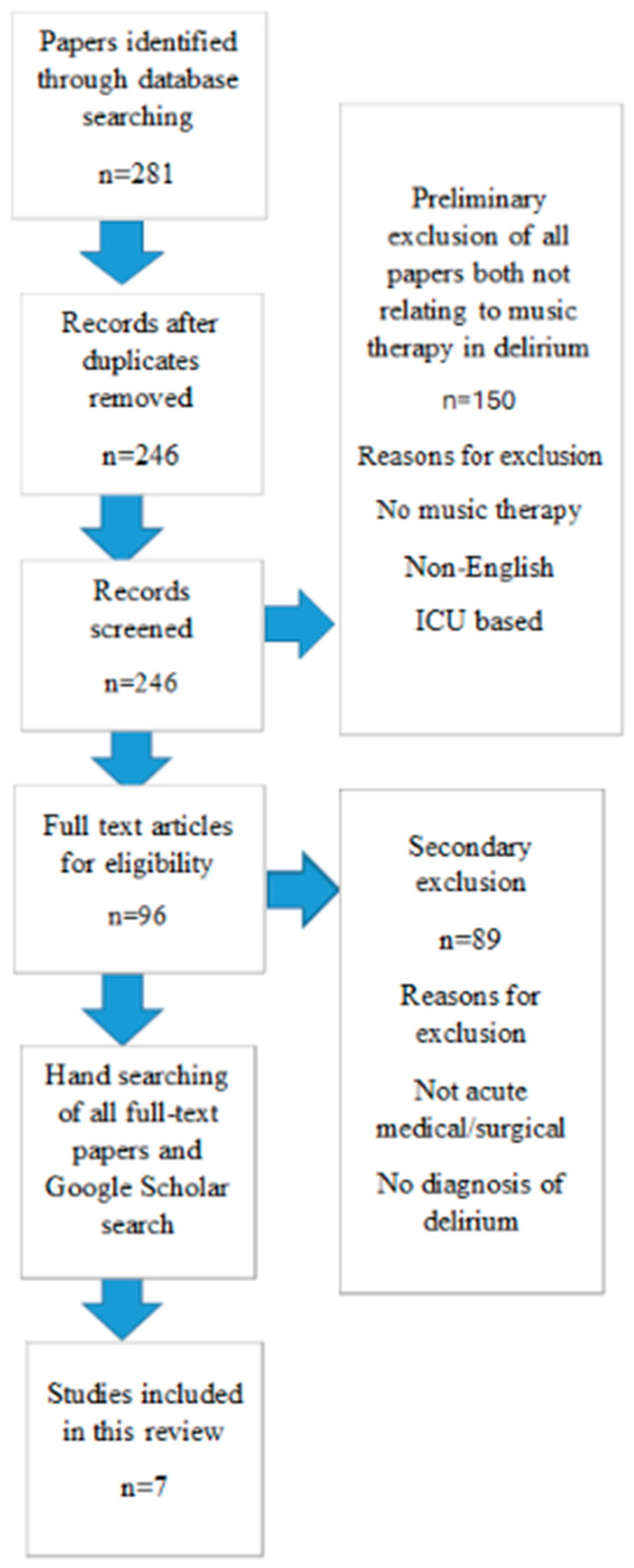
PRISMA flow diagram. PRISMA, Preferred Reporting Items for Scoping Reviews and Meta-Analyses.

The PRISMA flow diagram summarising the study selection process is presented in Figure 1.

### 3.1. Characteristics of Included Studies

Seven studies met the inclusion criteria for this review. All seven were quantitative, including five randomised controlled trials that examined the use of music therapy or structured music listening for adults with delirium or at risk of delirium during acute hospital admission [36,37,38,40,42]. Three postoperative studies by McCaffrey and colleagues demonstrated reductions in acute confusion, anxiety and pain among older adults following hip or knee surgery [36,37,38].

One non-randomised quantitative study evaluated Creative Music Therapy within a geriatric acute care unit and reported decreases in agitation and anxiety among older women with delirium or dementia [39]. A pilot randomised study in an emergency department assessed personalised music for older adults with suspected cognitive impairment and demonstrated good feasibility with potential benefits for delirium-related symptoms [40].

A feasibility randomised controlled trial compared personalised music with relaxing music in a geriatric ward and showed acceptable adherence with early signs of reduced delirium-related outcomes [41]. Another postoperative trial involving older surgical patients identified that structured music therapy reduced delirium incidence and improved sleep quality [42]. Of the included studies, only Cheong et al. [39] involved therapist-led Creative Music Therapy delivered by a trained music therapist; the remaining studies examined structured or personalised music listening interventions.

Across the seven studies, sample sizes ranged from 22 to 151, with a combined sample of 569 adults. Most participants were older adults, and several studies included a higher proportion of female participants. None of the studies reported ethnicity. Methodological quality was high overall, with six studies achieving the maximum Mixed Methods Appraisal Tool score and one study rating slightly lower due to minor issues with intervention adherence [Table 1].

### 3.2. Synthesis of Evidence

The included studies collectively demonstrate the multidimensional role of the therapeutic use of music for adults experiencing delirium in acute hospital settings. Although the interventions varied in format, duration and delivery, the studies consistently showed that music influences several domains relevant to delirium care, ranging from cognitive and behavioural outcomes to physiological stability and emotional well-being. For clarity, the term therapeutic use of music is used throughout the Results to encompass both therapist-led music therapy and structured music-based or music listening interventions, while the term music therapy is reserved exclusively for interventions delivered by a trained music therapist. From the narrative synthesis, three interlinked themes emerged: reductions in delirium-related symptoms, enhancements in physical recovery and improvements in psychosocial and emotional outcomes.

#### 3.2.1. Theme 1: Reduction in Delirium-Related Symptoms

The first and most prominent theme relates to changes in delirium-related symptoms following the therapeutic use of music. Three postoperative trials conducted by McCaffrey and colleagues examined recorded music listening interventions among older surgical patients [36,37,38]. These studies assessed outcomes including acute confusion, agitation and anxiety using validated psychological and behavioural measures, and each reported reductions in symptom scores following the intervention. Interventions were delivered as structured music listening during the perioperative period, with duration and frequency varying across studies. Together, these studies indicate that music listening was associated with improvements in selected delirium-related symptoms in postoperative populations, although study samples were small and outcomes were measured over short timeframes.

One study examined Creative Music Therapy delivered by a trained music therapist within a geriatric acute care unit [39]. This therapist-led, interactive intervention focused on relational engagement rather than passive listening and assessed outcomes including agitation, affect and restlessness using observational and behavioural measures. The study reported reductions in agitation and negative affect among older women with delirium or dementia following music therapy sessions.

Across the quantitative studies, a range of validated delirium and psychological assessment tools were used to measure outcomes, including instruments assessing confusion, anxiety and behavioural disturbance [36,37,38,39]. Two postoperative studies also measured preoperative anxiety, reporting reductions following music listening interventions [36,37]. These findings suggest that music-based interventions were associated with changes in anxiety-related outcomes in populations at risk of postoperative delirium. In contrast, the emergency department feasibility study examined brief exposure to personalised or relaxing recorded music and reported more modest symptom changes, alongside high acceptability and feasibility within a time-limited care context [40].

Overall, studies included within this theme reported improvements in delirium-related symptoms following music-based interventions, including reductions in confusion, agitation and anxiety. However, findings varied according to setting, intervention type, delivery format and exposure duration, and no conclusions regarding comparative effectiveness can be drawn. Collectively, this theme maps how delirium-related symptoms have been assessed and reported in relation to the therapeutic use of music in acute hospital settings [36,37,38,39,40,41,42].

#### 3.2.2. Theme 2: Music Therapy and Physical Recovery

While changes in delirium-related symptoms formed the dominant pattern, a second theme mapped outcomes relating to the therapeutic use of music and physical recovery processes relevant to delirium risk and management. Several postoperative studies examined recorded music listening interventions and reported reductions in pain intensity, analgesic requirements and self-reported physical comfort following exposure to music [36,37,38]. Pain outcomes were assessed using standard pain intensity measures, with intervention duration and frequency varying across studies. These findings indicate that music listening was associated with changes in pain-related outcomes in postoperative populations, although study samples were small and follow-up periods were limited.

Sleep-related outcomes were also reported. One randomised trial examined five-element music, delivered as a structured recorded music intervention rather than therapist-led music therapy, and assessed postoperative sleep quality using standard sleep measures [42]. This study reported improvements in sleep outcomes following the intervention. Similarly, a feasibility study conducted in acute geriatric wards compared personalised and relaxing recorded music and reported improvements in restfulness and reductions in nighttime agitation, based on observational and self-reported measures [41]. These studies indicate that sleep-related outcomes have been explored in relation to music-based interventions within acute hospital settings.

In contrast, one study examined Creative Music Therapy, delivered by a trained music therapist, and assessed outcomes related to alertness, engagement and participation in care [39]. This therapist-led intervention involved live, interactive music and reported increased patient responsiveness, emotional engagement and willingness to interact with staff following sessions. Some studies also explored physiological outcomes, including blood pressure and heart rate, although findings across these measures were variable and not consistently statistically significant [41,42].

Overall, this theme maps how pain, sleep, physiological parameters and engagement with care have been assessed in relation to the therapeutic use of music in acute hospital settings. Across studies, music-based interventions were associated with reported changes in these physical and behavioural outcome domains; however, variation in intervention type, delivery format, outcome measures and exposure duration limits comparison across studies and precludes conclusions regarding comparative effectiveness [36,37,38,39,40,41,42].

#### 3.2.3. Theme 3: Psychosocial and Emotional Outcomes

A third theme mapped psychosocial and emotional outcomes examined in relation to the therapeutic use of music among adults experiencing delirium in acute hospital settings. Across postoperative, emergency department and geriatric care studies, music-based interventions were explored in relation to outcomes including emotional distress, anxiety, mood and interpersonal engagement. In postoperative studies, music listening interventions assessed patient-reported emotional responses and anxiety-related outcomes, with participants reporting feeling calmer and more emotionally settled during periods of acute confusion [36,37,38]. These outcomes were primarily captured through self-report or observational measures over short intervention periods.

One study conducted in a geriatric acute care setting examined Creative Music Therapy, delivered by a trained music therapist, and assessed psychosocial outcomes including emotional expression, communication and mood states [39]. This therapist-led, interactive intervention involved live music engagement and reported increased emotional responsiveness and positive affect among older adults with delirium or dementia.

Interpersonal and relational outcomes were also described. A feasibility study in the emergency department examined personalised recorded music and assessed distress and patient–staff interaction, reporting reductions in observable distress and more cooperative engagement with healthcare providers [40]. Other studies described outcomes related to reminiscence, emotional grounding and recognition, particularly when personalised or culturally meaningful music was used [39,40,41]. These outcomes were primarily assessed through qualitative observations and descriptive reporting.

Overall, this theme maps how emotional regulation, interpersonal connection and patient experience have been examined in relation to music-based interventions in acute hospital contexts. Across studies, psychosocial outcomes were reported following both therapist-led music therapy and structured music listening interventions; however, variations in the study design, outcome measures and intervention delivery limits comparison and precludes conclusions regarding consistency or comparative effectiveness [36,37,38,39,40,41,42].

## 4. Discussion

This scoping review synthesised the current evidence on music therapy and music-based interventions for delirium in acute hospital settings. As a scoping review, these findings should be interpreted as a descriptive mapping of how music-based interventions have been studied and what outcomes have been reported, rather than as evidence of comparative effectiveness or causal impact. It extends existing literature by focusing specifically on non-ICU acute medical and surgical wards, highlighting an underexplored area of practice and identifying gaps relevant to nursing-led delirium care. The review also maps the therapeutic use of music, including structured music listening, as a non-pharmacological approach within acute hospital contexts. These findings should be considered within the broader understanding of delirium as a serious, multifactorial syndrome associated with high mortality, prolonged hospitalisation, cognitive decline and substantial distress for patients and families [43,44,45,46,47,48,49]. Despite long-standing international recommendations emphasising non-pharmacological approaches in delirium care [43,50,51,52,53,54], pharmacological agents such as antipsychotics and sedatives continue to be widely used, despite limited benefit and known risks [55,56,57]. In this context, there is a need to better understand the range of non-pharmacological strategies that have been explored in acute settings. Given the scoping nature of this review, findings should be interpreted as a descriptive mapping of how music-based interventions have been studied and what outcomes have been reported, rather than as evidence of comparative effectiveness.

Across the included studies, the therapeutic use of music, encompassing both therapist-led music therapy and structured music listening interventions, was examined in relation to delirium-related symptoms such as confusion, agitation and anxiety, particularly in postoperative populations [36,37,38,39]. These studies reported reductions in symptom scores following music-based interventions, using a range of validated behavioural and psychological measures. Proposed mechanisms discussed in the literature include modulation of arousal, attention and stress responses, which align with neurobiological models of delirium involving disrupted attention and sensory processing [58]. Several studies also reported reductions in preoperative anxiety [36,37], a factor recognised as contributing to delirium risk [52,54]. However, exposure duration, intervention delivery and outcome measurement varied across studies, and findings should be interpreted cautiously. Results from the emergency department feasibility study were more modest, likely reflecting brief intervention exposure within a time-limited care context [40].

The review also maps how music-based interventions have been explored in relation to physical recovery processes relevant to delirium risk and management. Postoperative studies reported changes in pain intensity, analgesic use and physical comfort following recorded music listening interventions [36,37,38], outcomes commonly associated with delirium vulnerability [52]. Sleep-related outcomes were also examined, with one randomised trial of five-element music and one feasibility study of personalised versus relaxing music reporting improvements in sleep or restfulness [41,42]. In addition, one therapist-led Creative Music Therapy study explored alertness, engagement and emotional responsiveness [39]. Although some physiological outcomes such as heart rate and blood pressure were also reported, findings were variable and not consistently statistically significant [41,42]. Collectively, these studies illustrate the range of physical and behavioural outcomes that have been examined, rather than providing evidence of consistent effects.

Psychosocial and emotional outcomes formed a further area of focus within the included literature. Music-based interventions were examined in relation to emotional distress, anxiety, mood and interpersonal engagement across postoperative, emergency and geriatric settings [36,37,38,39,40,41]. Studies reported patient perceptions of increased calmness, reassurance and emotional grounding during music listening, while therapist-led music therapy was associated with enhanced emotional expression and communication in older adults with delirium or dementia [39]. Several studies also described relational outcomes, including improved patient–staff interaction and evoked reminiscence, particularly when personalised or culturally meaningful music was used. These outcomes were primarily assessed through self-report or observational methods and varied across settings and intervention formats.

It is important to recognise that both therapist-led music therapy and structured music-based strategies have been explored within delirium care, with distinct but potentially complementary roles. Trained music therapists provide specialist assessment, clinical judgement and individualised therapeutic engagement; however, access to music therapy services in acute hospital settings may be limited or time-bound. The heterogeneity observed across study designs, intervention formats and outcome measures limits cross-study comparison and highlights the need for greater methodological consistency in future research. In this context, music-based strategies introduced or modelled by music therapists and subsequently used by patients, family members or healthcare staff may offer continuity of approach beyond formal therapy sessions, although further research is required to understand optimal implementation.

From a nursing practice perspective, the findings map how music-based strategies have been incorporated into routine delirium care within acute wards. Nurses are well positioned to identify patients who may benefit from music-based approaches, support person-centred music selection and integrate music into broader non-pharmacological delirium prevention bundles, such as sleep promotion, pain management and sensory orientation. Education and guidance are important to support safe and appropriate use, including attention to individual preferences and sensory thresholds. Where available, referral to trained music therapists remains important for patients with complex needs or where more interactive or relational interventions are indicated.

Ethically, music-based interventions were generally reported as acceptable and well-tolerated across studies, with no adverse events described when interventions were individualised and culturally appropriate [39,40,41]. This contrasts with the risks associated with pharmacological or restrictive approaches historically used to manage delirium-related agitation and aligns with person-centred principles of care.

The evidence base identified represents a progression from earlier reviews, which often included small, heterogeneous samples and limited methodological transparency [24,52]. Nonetheless, important challenges remain, including heterogeneity in intervention design, duration and outcome measurement, as well as limited representation of therapist-led music therapy studies. These limitations highlight the need for further well-designed research with clearer intervention reporting, longer follow-up and greater attention to context and implementation.

Overall, this scoping review maps the current evidence relating to music therapy and music-based interventions for adults experiencing delirium in acute hospital settings. The included studies report associations between the therapeutic use of music and outcomes related to delirium symptoms, physical recovery and psychosocial experience, although the evidence remains limited and heterogeneous. Future research should focus on strengthening methodological rigour, clarifying intervention components and exploring implementation within routine acute care practice.

### Strengths and Limitations

Strengths of this review include the use of a comprehensive and systematic search strategy across four major electronic databases (CINAHL, MEDLINE, PsycINFO and Embase), which ensured broad coverage of studies examining music therapy and music-based interventions in relation to delirium in acute hospital settings. The review followed established scoping review frameworks, including guidance from the Joanna Briggs Institute, Arksey and O’Malley, and the PRISMA-ScR standards [32,33,35], enhancing methodological transparency and reproducibility. By focusing on studies published within the last ten years, the review captured evidence most relevant to contemporary clinical practice. The inclusion of randomised controlled trials alongside non-randomised quantitative and feasibility studies allowed mapping of a range of clinical, psychological and behavioural outcome domains. The use of narrative synthesis was appropriate given the methodological and clinical heterogeneity of the included studies and supported integration of findings across diverse designs.

Several limitations should also be acknowledged. Most included studies examined music listening interventions rather than therapist-led music therapy, highlighting a need for further research led by qualified music therapists. Only English-language studies were included, meaning relevant evidence from non-English-speaking contexts may have been missed, potentially limiting the global applicability of findings. Although no geographical restrictions were applied, this language limitation may have contributed to over-representation of Western healthcare settings. The number of eligible studies was small, and several involved limited sample sizes or single-site designs, which constrains generalisability [36,37,38,39,40,41,42]. Considerable heterogeneity was present across studies in intervention format, timing, duration and outcome measurement, limiting direct comparison and precluding meta-analysis. Variation in delirium assessment tools further complicated synthesis, as some studies used validated delirium measures while others relied on broader behavioural scales. There is also a possibility of publication bias, as studies reporting favourable findings may be more likely to be published. In addition, none of the included studies reported participant ethnicity; while study country and care setting were extracted, limited information was available regarding whether cultural or individual music preferences informed intervention selection, which constrains interpretation and generalisability. Finally, this review focused specifically on acute hospital settings and therefore does not include evidence from long-term care or community settings, which were outside the scope of this review but may be relevant in other contexts.

## 5. Conclusions

This scoping review maps the existing evidence on music therapy and music-based interventions for adults experiencing delirium in acute hospital settings. Across the included studies, associations were reported between the therapeutic use of music and outcomes related to delirium-related symptoms, physical recovery processes and psychosocial experience. These findings suggest that music-based approaches have been explored as supportive, non-pharmacological strategies within acute care contexts, although the evidence base remains limited and heterogeneous.

The review indicates that both therapist-led music therapy and structured music-based interventions have been used alongside established non-pharmacological approaches such as sleep promotion, pain management, sensory orientation and family involvement. While findings across studies point to potential benefits for patient comfort, engagement and emotional regulation, conclusions regarding effectiveness or comparative impact cannot be drawn. Acceptance of music-based approaches among patients, families and healthcare staff was generally reported as favourable, supporting their feasibility within acute hospital environments.

While the existing evidence suggests that music-based approaches have been explored as supportive, low-risk strategies within acute delirium care, further well-designed studies are required to clarify optimal intervention characteristics, implementation processes and outcome measurement within routine acute hospital practice. Future studies should also examine implementation processes, contextual factors and the specific contribution of therapist-led music therapy. As healthcare systems continue to prioritise person-centred, low-risk approaches to delirium care, understanding how music-based interventions can be appropriately integrated into routine acute practice remains an important area for ongoing investigation.

## Figures and Tables

**Table 1:** A summary of the characteristics of the included studies.

Paper	Author	Design	MMAT Score
1	McCaffrey et al. [36]	Quantitative RCT	5
2	McCaffrey et al. [37]	Quantitative RCT	5
3	McCaffrey [38]	Quantitative RCT	5
4	Cheong et al. [39]	Quantitative non-RT	5
5	Keene et al. [40]	Quantitative RCT	4
6	Golubovic et al. [41]	Quantitative Descriptive	5
7	Han et al. [42]	Quantitative RCT	5

## Data Availability

The datasets generated and analysed during the current study are available from the corresponding author on reasonable request.

## Supplementary Materials

The following supporting information can be downloaded at: https://www.mdpi.com/article/10.3390/nursrep16010023/s1, Supplementary File S1. Search strategy for the scoping review.

## Public Involvement Statement

No public involvement in any aspect of this research.

## Guidelines and Standards Statement

This scoping review was conducted and reported in accordance with the Preferred Reporting Items for Systematic Reviews and Meta-Analyses Extension for Scoping Reviews (PRISMA-ScR) guideline, which provides a structured framework for the transparent reporting of scoping review methodology and findings [35].
