# Supplementary material for: Effectiveness of Music Therapy for Delirium in Acute Hospital Settings: A Scoping Review
Source: Nurs Rep. 2026 Jan 12;16(1):23. doi: 10.3390/nursrep16010023 (PMC12844646; doi:10.3390/nursrep16010023)
Supplement: Supplementary file 1 [file nursrep-16-00023-s001.zip › nursrep-4040815-supplementary.pdf]

Supplementary File S1 – MDPI Music Therapy in Delirium

Search overview

Electronic searches were conducted in October 2024 in CINAHL, MEDLINE (Ovid), PsycINFO, and Embase. Searches combined controlled vocabulary and free-text terms relating to delirium and the therapeutic use of music. Searches were conducted in title and abstract fields where applicable. No date limits were applied at the database search stage; publication date restrictions were applied during eligibility screening. Search strategies were adapted for each database.

CINAHL

1. delirium OR confusion OR acute confusion OR mental confusion OR altered mental status
2. music therapy OR musical intervention OR music based therapy OR music treatment OR music assisted therapy OR sound therapy OR auditory-verbal therapy
3. 1 AND 2

Notes on search sensitivity and terminology

Broad delirium-related terms were intentionally included to maximise sensitivity and capture studies where delirium was described using alternative terminology. Music-related terms were mapped to controlled vocabulary where available and supplemented with free-text terms to address inconsistent labelling of music therapy and music-based interventions in the literature. Adjacency operators were not used.
